# Effective gamma-ray sterilization and characterization of conductive polypyrrole biomaterials

**DOI:** 10.1038/s41598-018-22066-6

**Published:** 2018-02-27

**Authors:** Semin Kim, Jin-Oh Jeong, Sanghun Lee, Jong-Seok Park, Hui-Jeong Gwon, Sung In Jeong, John George Hardy, Youn-Mook Lim, Jae Young Lee

**Affiliations:** 1 School of Materials Science and Engineering, Gwangju Institute of Science and Technology, Gwangju, 61005 Republic of Korea; 2 Research Division for Industry & Environment, Advanced Radiation Technology Institute, Korea Atomic Energy Research Institute (KAERI), 29 Gumgugil, Jeongeup, 56212 Republic of Korea; 3 Materials Science and Engineering Concentration, GIST College, Gwangju Institute of Science and Technology, Gwangju, 61005 Republic of Korea; 4 Department of Chemistry, Lancaster University, Lancaster, Lancashire LA1 4YB United Kingdom; 5 Materials Science Institute, Lancaster University, Lancaster, Lancashire LA1 4YB United Kingdom; 6 Department of Biomedical Science and Engineering, Gwangju Institute of Science and Technology, 500-712 Republic of Korea, Gwangju, 61005 Republic of Korea

## Abstract

Conductive polymers, including polypyrrole (PPy), have been extensively explored to fabricate electrically conductive biomaterials for bioelectrodes and tissue engineering scaffolds. For their *in vivo* uses, a sterilization method without severe impairment of original material properties and performance is necessary. Gamma-ray radiation has been commonly applied for sterilization of medical products because of its simple and uniform sterilization without heat generation. Herein we describe the first study on gamma-ray sterilization of PPy bioelectrodes and its effects on their characteristics. We irradiated PPy bioelectrodes with different doses (0–75 kGy) of gamma-rays. Gamma-ray irradiation of the PPy (γ-PPy) increased the oxygenation and hydrophilicity of the surfaces. Interestingly, gamma-ray irradiation did not alter the electrical impedances and conductivities of the PPy substrates. Additionally, γ-PPy prepared with various dopants (e.g., para-toluene sulfonate, polystyrene sulfonate, and chlorine) showed the electrochemical properties similar to the non-irradiated control. Gamma-ray irradiation at doses of ≥15 kGy was required for effective sterilization as evidenced by complete eradication of gram positive and negative bacteria. γ-PPy substrates also showed cytocompatibility similar to untreated control PPy, indicating no substantial alteration of cytocompatibility. In conclusion, gamma ray sterilization is a viable method of sterilization of conducting polymer-based biomaterials for biomedical applications.

## Introduction

Conducting polymers (CPs) have attracted attention from scientists and engineers due to their high electrical conductivity, simple synthesis, and excellent biocompatibility^1,2^. CPs have also been extensively explored to fabricate electrically conductive biomaterials, which allow for efficient delivery of electrical signals with low electrochemical impedance, high charge injection capability, and biocompatibility^3,4^, for bioelectrodes and tissue engineering scaffolds applications^1,5,6^. Commonly used CPs include poly(3,4-ethylenedioxythiophene) (PEDOT), polyaniline (PANi), polythiophene (PT), and polypyrrole (PPy). CPs are recognized as promising materials for the development of neural prosthetics^7–10^, cochlear implants^5,11^, drug delivery devices^12,13^, bioactuators^14,15^, and biosensors^4,16,17^.

To clinically translate CP-based biomaterials including bioelectrodes for implantation in the body, effective sterilization methodologies need to be established which eradicate bacterial infection and ensure their biocompatibility. Sterilization of CP-based biomaterials for *in vivo* uses is challenging because it may lead to the deterioration of the CP’s inherent electrical and biochemical properties, and there is a lack of systematic studies validating the effects of sterilization methods on the properties of such CP-based biomaterials. Conventional sterilization methods, such as steam, ultraviolet radiation (UV), ethylene oxide (EO), and gamma-irradiation sterilization, can be employed for CPs^18,19^. Among them, sterilization by gamma-ray irradiation offers several important advantages, including: gamma-rays can easily and uniformly reach all parts of the object to be sterilized; sterilization can be performed with different doses for various materials (e.g., heat-sensitive materials) in various environments (e.g., at low temperatures in gas, solid, or liquid states)^20–22^. However, irradiation with high-energy gamma-ray can cause various chemical reactions in polymers, including polymer chain scission, crosslinking, and degradation^23–26^. An excessive dose of gamma-ray can lead to the alteration of material’s chemical, electrical, mechanical and biological properties. The focus of this study is to understand the effects of gamma-ray irradiation on both sterilization effectiveness and materials properties of PPy bioelectrodes, which are of critical importance for their eventual translation.

**Figure 1:**
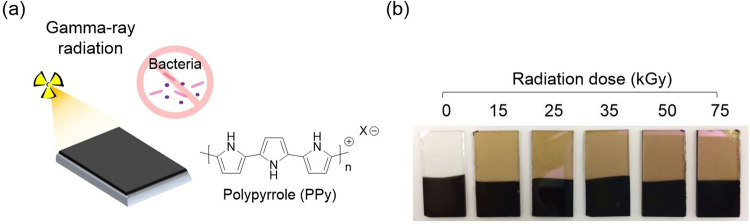
(**a**) A schematic illustration of gamma-ray sterilization of PPy biomaterials. (**b**) Photographs of the PPy-deposited ITO electrodes after exposure to different doses of gamma-rays.

In this study, we synthesized para-toluene sulfonate (pTS)-doped PPy (PPy/pTS) coated electrodes and exposed them to various doses of gamma-rays to study the suitability of gamma-ray radiation for the sterilization of CP-based bioelectrodes (Fig. 1). PPy has been widely studied for biomedical applications (e.g., electrodes, biosensors, and tissue engineering scaffolds) due to its facile synthesis, high electrical conductivity, excellent stability, and good biocompatibility^27–31^. We carefully characterized the chemical, electrochemical, and biological properties of the irradiated PPy/pTS (γ-PPy) electrodes using a variety of methods. In addition, the effectiveness of sterilization via gamma-ray irradiation was assessed with gram positive and gram negative bacteria. Finally, the cytocompatibility of the γ-PPy substrates was studied using various types of cells (e.g., neuronal cells, myoblasts, and fibroblasts).

## Results

### Electrochemical fabrication of PPy electrodes and gamma-ray irradiation

A PPy/pTS was electrochemically polymerized on ITO or gold electrodes (Supplementary Fig. S1), followed by exposure to gamma-rays. The PPy/PSS substrates irradiated with different radiation doses (0, 15, 25, 35, 50, and 75 kGy) were denoted as γ-PPy *x*, in which *x* indicates a dose (kGy). After gamma ray irradiation, the ITO-glass changed color from transparent to light brown (Fig. 1b). The various γ-PPy samples were characterized via a variety of techniques as shown in the following results.

### Characterization of gamma-irradiated PPy electrodes

**Figure 2:**
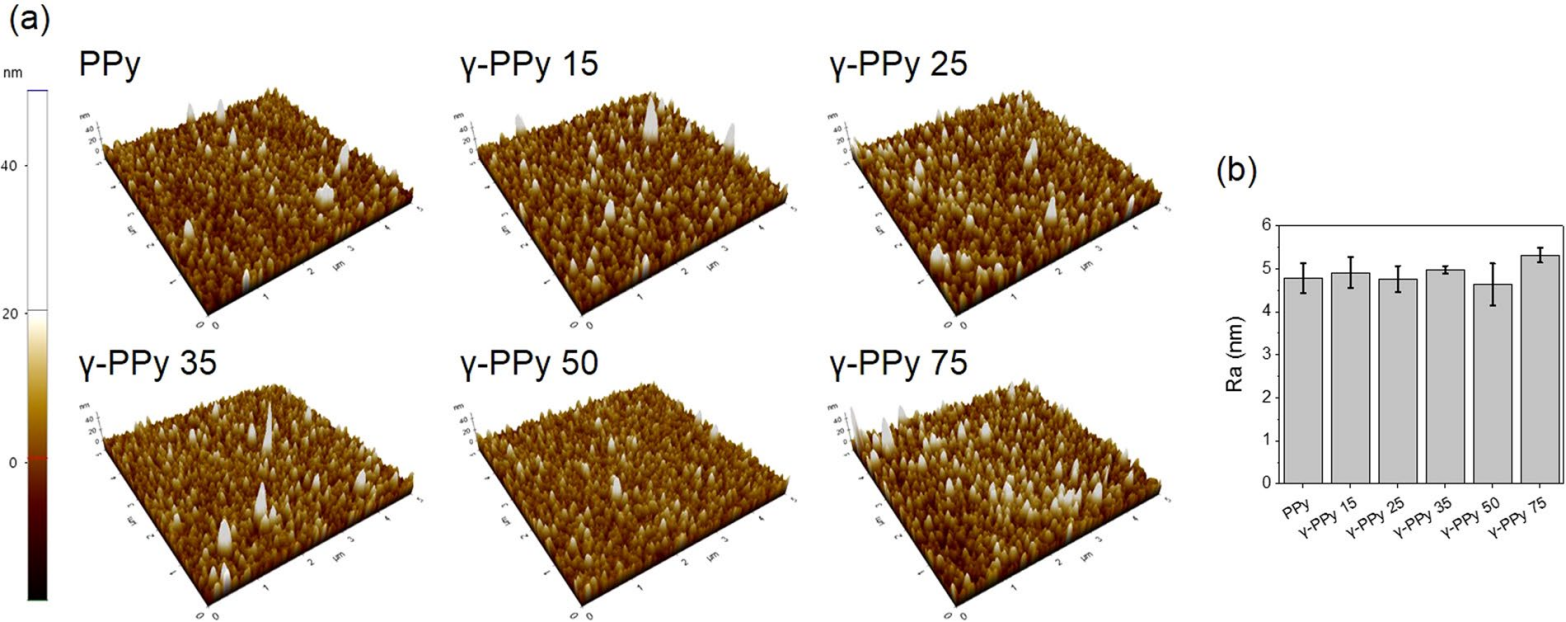
(**a**) Atomic force micrographs of PPy and γ-PPy samples irradiated with different doses of gamma-ray. (**b**) Average roughness (root mean square) of PPy and γ-PPy samples.

The surface morphologies of the PPy and γ-PPy were analyzed by AFM. As shown in Fig. 2, both PPy and various γ-PPy exhibit similar surface features with numerous spherical nodules with grains of similar sizes (100–200 nm) analogous to those reported by other researchers^32^. The surface roughness was not significantly different between PPy and γ-PPy. Furthermore, SEM images of PPy and γ-PPy confirm that gamma-ray irradiation does not influence the morphology of the PPy surfaces (Supplementary information Fig. S2).

**Figure 3:**
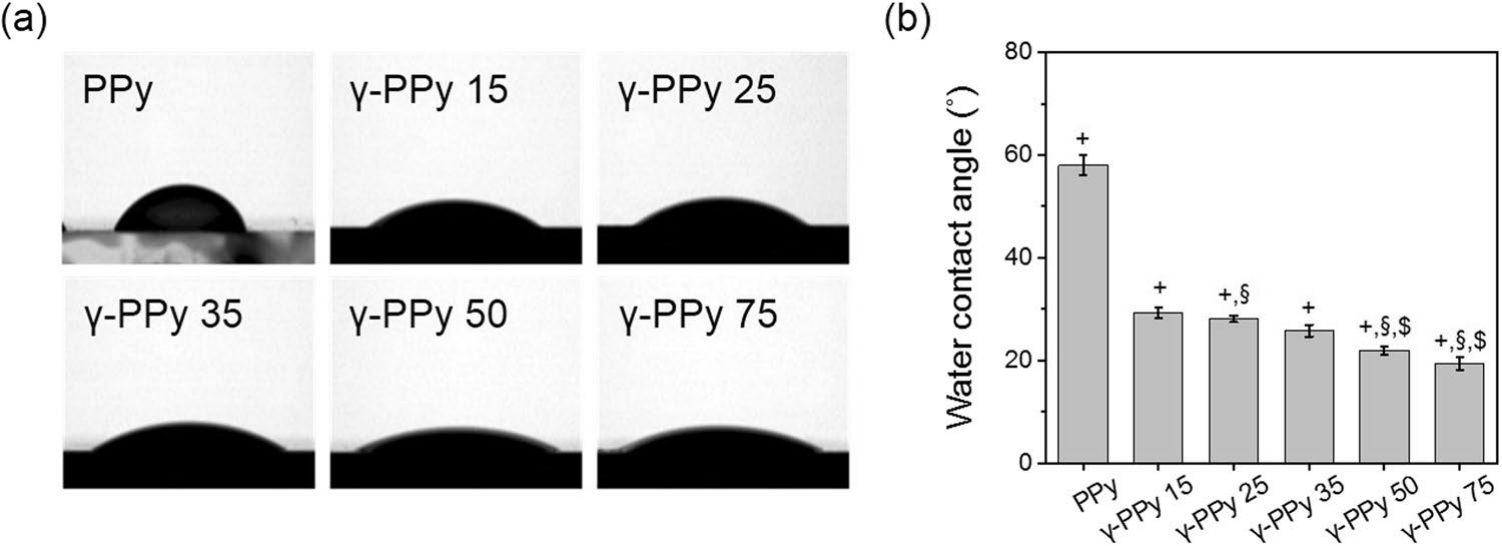
Water contact angle measurement. (**a**) Photographs of water droplets on PPy and γ-PPy electrodes. (**b**) A plot of water contact angles (*n* = 3, ^+^p < 0.05 versus PPy, ^§^p < 0.05 versus γ-PPy 25, ^$^p < 0.05 versus γ-PPy 25.

Gamma-ray radiation increased the hydrophilicity of the PPy samples (Fig. 3). Hydrophilicity of the PPy surfaces increased with the increased doses of gamma-ray from 15 to 75 kGy. Non-irradiated PPy had a water contact angle of 58.04 ± 1.92°, whereas those of γ-PPy irradiated with 15, 25, 35, 50, and 75 kGy were 29.33 ± 1.02, 28.06 ± 0.62, 25.74 ± 1.15, 21.90 ± 0.73, and 19.35 ± 1.26°, respectively. Compared to the PPy, the γ-PPy 75 had approximately 40° smaller contact angles.

**Figure 4:**
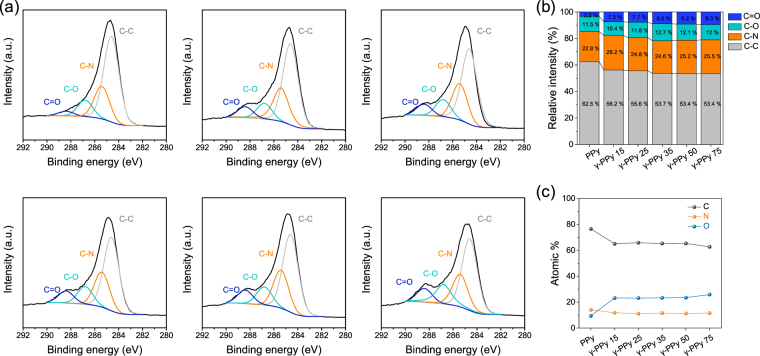
XPS spectra of PPy and γ-PPy surfaces. (**a**) High-resolution C_1s_ spectra with deconvoluted peaks from the PPy and γ-PPy. (**b**) Relative intensities of carbon peaks in the spectra. (**c**) Relative composition of carbon, nitrogen, and oxygen atoms on the surfaces.

The chemical compositions of γ-PPy surfaces were analyzed by XPS and compared with that of non-irradiated PPy (Fig. 4). High-resolution C_1s_ spectra of the PPy and γ-PPy films were deconvoluted into four peaks, which included 284.6 (C-C/C-H), 285.5 (C-N), 286.8 (C-O), and 288.4 eV (C=O), according to the literatures^9^. With an increase in gamma-ray dose, the intensities of the peaks at 286.9 eV (C-O) and 288.4 eV (C=O) increased. The sum of relative intensities of these oxygenated carbons (C-O and C=O) were 14.7% for PPy, whereas γ-PPy-75 had 21.3% of these oxygenated carbon peaks, respectively. Consequently, gamma ray irradiation caused more oxygenation on the PPy surfaces. Ozone and oxygen radicals would be generated during gamma-ray irradiation which reacts with the oxygen and/or water molecules in the air, which oxidises the PPy, which is supported by surface elemental analysis indicating more oxygen atoms on the PPy electrodes irradiated with the higher doses.

The adhesion of PPy coatings on the electrodes after gamma-ray irradiation was examined by using the Scotch tape detachment test (Supplementary information Fig. S3). The pristine PPy coating was easily detached from the ITO electrode by scotch tape. For the γ-PPy, the PPy coating was stronger with higher doses of gamma-ray, implying the contribution of gamma-ray for the formation of stronger intermolecular interactions between the electrode surfaces and PPy films.

### Electrochemical and electrical properties of the γ-PPy

**Figure 5:**
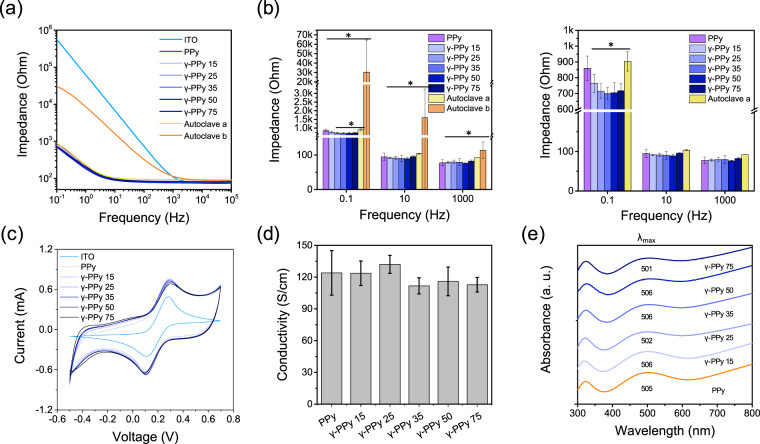
Characterization of electrochemical and electrical properties of the electrodes. (**a**) Bode plots and (**b**) Impedances at 0.1, 1, and 10^3^ Hz of various electrodes. Ten PPy electrodes were autoclaved. Among them, five electrodes showing the lowest impedances were selected and noted as ‘*Autoclave a*’ and all the 10 autoclaved electrodes were noted as ‘*Autoclave b*’ in the plots. (**c**) Cyclic voltammograms of the electrodes in a presence of 5 mM [Fe(CN)_6_]^3−/4−^ in 0.1 M KCl solution at a 0.1 V/s scan rate. (**d**) Conductivity of PPy and γ-PPy. (**e**) UV/vis spectra of PPy and γ-PPy electrodes.

Electrochemical impedance spectroscopy (EIS) was employed to evaluate the electrical/electrochemical properties of the PPy electrodes before and after gamma-ray irradiation. Possible alteration of the electrical properties of electrodes after sterilization should be carefully examined to ensure their inherent electrode performances. The impedances of γ-PPy electrodes were similar to those of non-irradiated PPy over the frequencies tested (Fig. 5a,b). These results suggest that gamma-ray irradiation with 15–75 kGy doses did not impair the electrode’s original electrochemical properties. For comparison, we autoclaved the PPy electrodes and measured the EIS. We tested 10 autoclaved samples (Supporting Information Fig. S4), finding that the averages of the impedances of all the autoclaved PPy electrodes (noted as ‘*Autoclave b’*) showed higher impedances than pristine PPy electrodes or gamma irradiated PPy electrodes. Five of the 10 autoclaved electrodes showed relatively good conductance, which was noted as ‘*Autoclave a*’ in the Fig. 5b. The reason of high increases in impedances of autoclaved PPy electrodes (5 out of 10) was attributed to local detachment of the PPy films from the ITO electrodes (Supporting Information Fig. S5), which occurs during autoclaving the samples due to differences in the thermal expansion of PPy and ITO. In addition, cyclic voltammetry indicates that the redox peaks, peak potential separation and capacitance of γ-PPy electrodes were not significantly changed by gamma-ray radiation (Fig. 5c, Supporting Information Fig. S6). The conductivity of the γ-PPy was not significantly different from the non-irradiated PPy, suggesting no impairment of inherent molecular structures or electronic properties of PPy after gamma-ray irradiation (Fig. 5d). UV-vis spectroscopy was employed to examine the changes in electronic energy states of γ-PPy after gamma-ray radiation (Fig. 5e). No significant shift of the peaks corresponding polaron-biopolaron transition at 500–510 nm was observed for the γ-PPy samples. These peaks are indicative of lengths of PPy chains and conduction bands^33^, which suggests that the electronic states and conjugation structures of PPy were stable to the doses of gamma-ray irradiation to which they were exposed.

**Figure 6:**
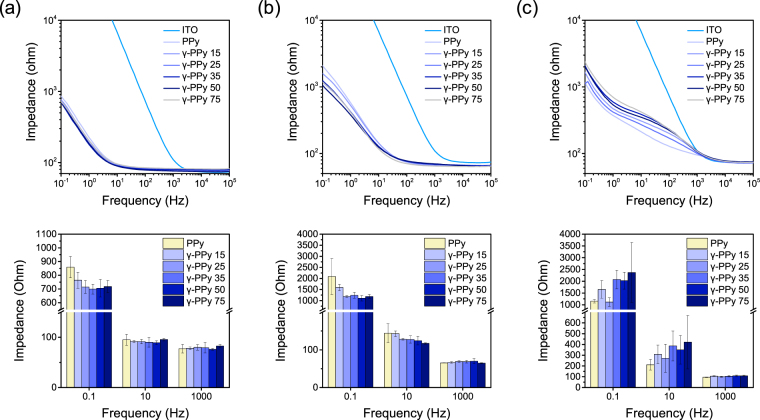
Electrochemical properties of PPy electrodes prepared with different dopants, (**a**) PPy/pTS, (**b**) PPy/PSS, (**c**) PPy/Cl, after the gamma-ray irradiation with different doses.

Moreover, we explored the possible effects of dopants on changes in electrochemical properties of PPy after gamma-ray irradiation (Fig. 6). Three different dopants were used for the preparation of PPy-electrodes, which included PPy/pTS, PPy/PSS, and PPy/Cl. The radiation did not significantly affect impedances of PPy/PSS at all frequency ranges, which was similar to the results of pTS-doped PPy (PPy/pTS). For γ-PPy/Cl, impedances slightly increased after irradiation; however, these differences were not significant. The results imply that gamma-ray irradiation can be employed for different PPy-based biomaterials doped with various dopants with minimal deterioration of the electrochemical characteristics of the PPy electrodes.

### Sterilization assay

**Figure 7:**
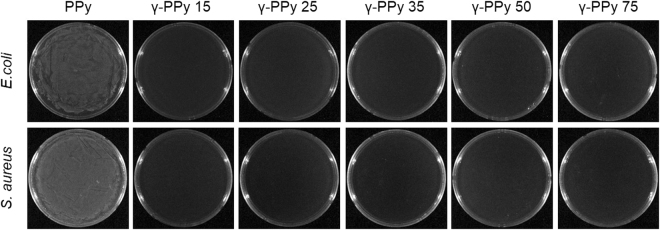
Sterilization test of the PPy irradiated with different doses of gamma-ray irradiation. Photographs were acquired 24 h after seeding the medium incubated with the irradiated PPy samples. After seeding the agar medium was incubated at 37 °C for 18 h.

Sterilization of PPy by gamma-ray was studied using the PPy electrodes in *E. coli* and *S. aureus* containing medium. After the gamma-ray irradiation (15, 25, 35, 50, and 75 kGy), the irradiated PPy electrodes were further incubated in the LB medium at 37 °C for 18 h to enrich possible bacteria that maintained their viability during the gamma-ray radiation (Supporting Information Fig. S7). As shown in Fig. 7, neither *E. coli* nor *S. aureus* was detected from the incubated solution. Complete bacterial eradication was observed for all the γ-PPy samples after irradiation at 15, 25, 35, 50, and 75 kGy. The results indicate that gamma-ray irradiation (≥15 kGy) is a valid methodology for sterilization of PPy-based biomaterials.

### Cytocompatibility tests of the γ-PPy

**Figure 8:**
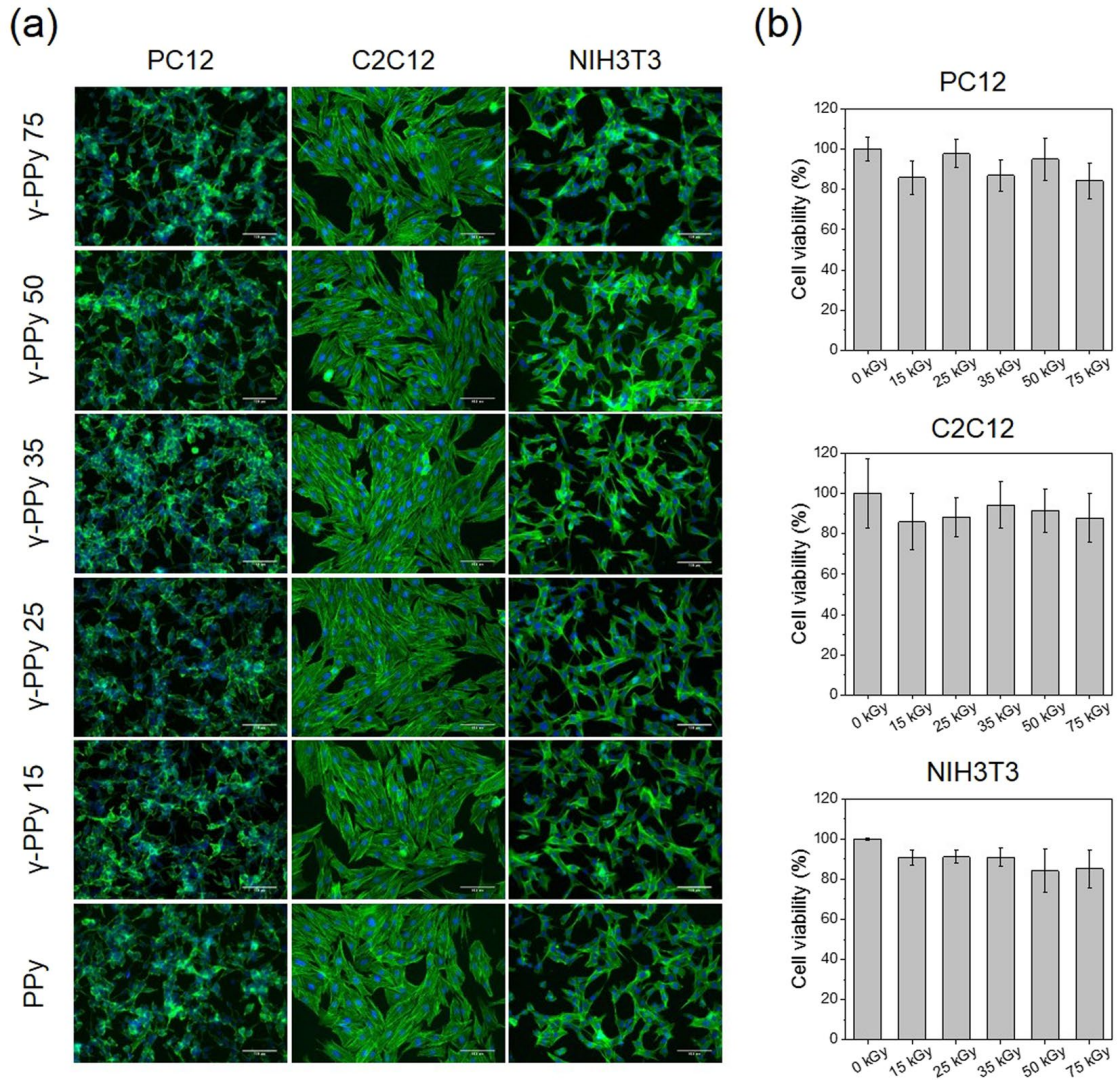
Cytocompatibility test of γ-PPy. (**a**) Immunofluoresence images of PC12, NIH3T3, and C2C12 cultured on PPy and various γ-PPy for 48 h. Cells were stained for *F*-actin. Scale bars are 100 μm. (**b**) Viability of the various cells cultured on PPy and γ-PPy *n* = 3.

Three different cell types, including PC12 neuronal cells, C2C12 myoblasts, and NIH3T3 fibroblasts, were cultured to investigate the cytocompatibility of the various γ-PPy samples and possible changes in their cellular interactions. In particular, these cells were selected to demonstrate the potential uses of gamma-ray sterilized PPy biomaterials for various biomedical applications, such as electromyography^34^ and electroencephalography^35^, and electrically conductive scaffolds for skeletal muscle^36^, neural^37^, or other tissue regeneration^38^. As shown in Fig. 8a, cells adhered well and spread on the all of the samples (non-irradiated PPy and irradiated PPy), indicating gamma-ray irradiation at the doses of 15–75 kGy did not influence overall cellular interactions. In case of PC12 cells, the cells were differentiated by forming multiple neurites on both PPy and γ-PPy. Good adhesion, growth, and neuritogenesis of PC12 cells on γ-PPy were compatible with pristine PPy (non-irradiated PPy). Likewise, γ-PPy supported adhesion and growth of C2C12 and NIH3T3 cells similarly to the non-irradiated PPy. All types of cells tested in this study displayed well-spread morphologies both on the PPy and γ-PPy electrodes. WST-1 assay was performed to quantify the viability of the cells growing on various PPy and γ-PPy. As shown in Fig. 8b, the viability of the PC12 cells, C2C12 myoblasts, and NIH3T3 cell on PPy and γ-PPy was not significantly different. These results indicate that sterilization using gamma-ray irradiation does not diminish the cytocompatibility of PPy-based biomaterials (and cellular interactions with γ-PPy is similar to the pristine PPy).

## Discussion

CP-based biomaterials are an emerging class of materials that efficiently mediate electrical signals with biological tissues and have a variety of potential applications^8^. Knowledge that electrical signals play pivotal roles in biological functions (such as electrophysiology and regeneration of damaged tissues^39^) has encouraged the development of CP-based biomaterials for various purposes such as bioelectrodes and tissue engineering scaffolds^40^. Bacterial infection is one of the most important issues related to the successful clinical translation of biomaterials because bacterial infections can lead to severe diseases for patients and/or malfunction of the implants^41^. In particular, bacterial contamination of bioelectrodes causes biofilms on the electrode surfaces, which is known as bio-fouling^42^. Bacterial infection and biofilm formation on electrodes does not only induce the inflammatory responses in the body but also leads to the formation of an electrically non-conductive layer on electrode surfaces, which results in the impairment of electrical and electrochemical performances of the bioelectrodes^43^. Therefore, sterilization of electrodes is essential for their biocompatibility and performance. To achieve manufacturing scale sterilization of biomaterials and biodevices, conventional sterilization methods such as steam, ultraviolet radiation (UV), ethylene oxide (EO), and gamma-irradiation sterilization can be employed for CP-based biomaterials^18,19^. The EO sterilization is widely used due to low temperature process and affordability; however, the residual toxic EO gas needs to be removed after the sterilization and thus the sterilization process requires longer time to complete the processes^44,45^. Steam sterilization, despite commonly employed, can cause thermal damages to CP characteristics and requires special processing to pack materials. Recently, Malliaras and colleagues studied the characteristics of PEDOT/PSS devices before and after autoclave at 121 °C for 20 min. They found that the PEDOT:PSS devices exhibited slightly increased impedance after the steam sterilization^19^. We also found that autoclave of PPy/pTS led to dramatic increases in impedance (in part attributable to cracks and local detachment of the PPy coatings), and five out of ten PPy electrodes lost their original electrochemical activities. On the other hand, sterilization by gamma-irradiation provides many advantages over steam, UV, or EO sterilization methods (e.g. simplicity and effectiveness). Our results indicate that the gamma-ray irradiation with at least 15 kGy can eliminate the gram positive and gram negative bacteria (*E. coli* and *S. aureus*).

Irradiation with gamma-rays led to increases in hydrophilicity of PPy due to the generation of polar oxygenated carbons (i.e., C-O and C=O) on the surface of the PPy as evident by XPS analysis. Wetting of CPs can play a positive role in electrochemical functions as the wetting of the CPs can benefit conductance of charge transfer at the CP surfaces^46^. Hydrophilic surfaces can hinder non-specific adsorption and denaturation of proteins, which can cause inflammatory reactions *in vivo*^47^. Furthermore, bonding of PPy films on electrodes was found to be greatly improved after irradiation. CP-coatings on electrode surfaces are usually not strongly adherent and often delaminated^48^. It should be noted that autoclaves cause the delamination of the films and crack formation. Hence, enhanced adhesion of γ-PPy on electrodes is an important benefit to ensure *in vivo* performances of the PPy bioelectrodes.

We originally expected that high energy of gamma-ray could detract the electrical properties of PPy because high energy gamma-ray can cause degradation of polymer chains and intermediate molecules (e.g., oxidants) generated during the radiation might damage the chemical structures and electrical performance of the PPy-based bioelectrodes. Surprisingly, gamma-ray irradiation with the doses of 15–75 kGy did not critically influence the electrical properties of the PPy electrodes. Our conductivity measurements and UV/vis spectral analyses of the γ-PPy revealed no significant changes in the electronic states of γ-PPy, indicating minimal structural damages by PPy during gamma-ray irradiation (Fig. 5). In addition, gamma-ray irradiation of differently doped PPy (e.g., PPy/pTS, PPy/PSS, and PPy/Cl) resulted in no substantial changes in impedances, indicating that gamma-rays can be used to sterilize PPy-based biomaterials doped with various substances.

The cytocompatibility of γ-PPy was tested to ensure the sterilized PPy was cell adherent opening possibilities for applications using neuronal cells (PC12), myoblasts (C2C12), and fibroblasts (NIH3T3). The cellular morphologies and adhesion were not significantly different among PPy and γ-PPy samples, indicating the good cytocompatibility of γ-PPy. Consequently, we demonstrated that gamma-ray irradiation is suitable for sterilization of PPy-based biomaterials as their cytocompatibility and support for the adhesion and growth of various types of cells are preserved even after the gamma-ray irradiation.

## Conclusion

We aimed to examine the impacts of the gamma-ray sterilization on conducting polymer (i.e., PPy)-based biomaterials. We show that PPy electrodes inoculated with *E. coli* and *S. aureus* are effectively sterilized using gamma-ray irradiation at doses of ≥15 kGy. The gamma-ray sterilization does not change the surface morphology of the PPy substrates, yet slight oxidation and increased hydrophilicity of PPy were observed in a dose dependent manner. Steam sterilization often caused instability of the PPy films and dramatically increased impedance; however, gamma-ray sterilization did not impair electrical and electrochemical properties of the PPy electrodes with the doses of 15–75 kGy. *In vitro* cell culture studies demonstrated that gamma-ray radiation did not deter cell adhesion on the substrates. We believe that gamma-ray sterilization for CP-based biomaterials can be utilized for a variety of interesting biomedical applications (including implantable electrodes, biosensors, prosthetics, and tissue engineering scaffolds).

## Methods

### Electrochemical fabrication of PPy/pTS electrodes

Pyrrole (Sigma-Aldrich) was purified by passing it through aluminum oxide (Sigma-Aldrich) column before use. The polymerizing solution for PPy/pTS was prepared with 0.15 M pyrrole and 0.1 M para-toluene sulfonate (pTS) (Sigma-Aldrich) in deionized (DI) water. For the preparation of PPy/PSS and PPy/Cl, the solutions of 0.1 M sodium polystyrene sulfonate (NaPSS) (Sigma-Aldrich) and 0.1 M sodium chloride (NaCl) (Sigma-Aldrich) were prepared with 0.15 M pyrrole, respectively. A PPy film was electrodeposited galvanostatically on indium-tin oxide (ITO) coated glass electrodes or gold electrodes using the three-electrode system consisting of a VersaSTAT3 electrochemical working station (Princeton Applied Research, AMEMEK Scientific Instrument, Berwyn, USA). A platinum wire electrode was used as a counter electrode, and a saturated calomel electrode (SCE, CH Instrument, Inc., TX, USA) was used as a reference electrode. The current density of 1 mA/cm^2^ was applied to the electrolyte solution. Charges of 50 mC/cm^2^ or 100 mC/cm^2^ were employed for the electrochemical PPy deposition. The polymerized PPy films were then carefully washed with DI water.

### Gamma ray irradiation of PPy electrodes

Prior to gamma-irradiation, the PPy-coated ITO electrodes were placed in a 6 well plate. The plates containing the electrodes were packed in aluminum pouches. Then, gamma 60-Co source (ACEL type C-1882) was irradiated to the samples with different radiation doses (15, 25, 35, 50, and 75 kGy) at a dose rate of 10 kGy/h. The gamma 60-Co source was located at the Korea Atomic Energy Research Institute (KAERI), Jeongeup, Republic of Korea.

### Steam sterilization (Autoclave)

Steam sterilization of the PPy-deposited electrodes was performed using autoclave (Hanbaek Scientific Co., Republic of Korea). The samples were placed into autoclave pouches (Duo-Check® Sterilization Pouch, Crosstex International) and then autoclaved at 121 °C for 20 min.

### Surface topography analysis

The surface morphologies of PPy and γ-PPy were explored using a field emission scanning electron microscope (FE-SEM, S-4700, Hitachi, Tokyo, Japan) with an electron beam of 10 kV and a working distance of 12.2 mm. The samples were not coated with metals because of their conductive properties. The surface topography and roughness of the PPy and γ-PPy electrodes were analyzed by atomic force microscopy (AFM, XE-100, Park System, Seoul, Republic of Korea). The experiments were performed using an NCHR tip (NCHR Nanoworld, Neuchâtel, Switzerland). All images were acquired at a 0.3 Hz scan rate in tapping mode.

### Water contact angle measurement

The water contact angles of the PPy and γ-PPy were measured using a Phoenix 300 contact angle analyzer (Surface Electro Optics Co., Suwon, Republic of Korea) according to the static sessile drop method. Distilled water (10 µL) was dropped on three or more positions on the electrodes. Experiments were performed in triplicate.

### X-ray photoelectron spectroscopy

Surface elemental analysis was performed by X-ray photoelectron spectroscopy (XPS, VG Multilab 2000 spectrometer, Thermo Scientific, Waltham, USA) equipped with an Al Kα X-ray source at 1486.6 eV which is monochromatic. High resolution carbon 1 s (C_1s_) spectra were further deconvoluted using the XPS PEAK software (University of Hong Kong, Hong Kong).

### Electrochemical impedance spectroscopy (EIS)

The electrochemical impedance spectra of the PPy and γ-PPy electrodes were obtained using VersaSTAT3 electrochemical working station with a three-electrode setup. Tape with a circular hole (diameter of 6 mm) was attached onto the electrode surfaces to ensure the same exposed electrode areas (0.2829 cm^2^) in all samples. Impedance measurement of the electrodes was performed in the presence of redox probe solution (5 mM [Fe(CN)_6_]^3−/4−^ (Sigma-Aldrich) in PBS) with an amplitude of 5 mV in a range of 0.1–10^5^ Hz.

### Conductivity measurement

The electrical conductivity of the PPy and γ-PPy was measured using a four-point probe method (Modysystems, Korea) that the linear scan voltammetry was applied from −1 V to 1 V. The PPy film thickness was measured using a stylus profiler (Dektak XT, Bruker) and resistance was measured from the *I*–*V* curve. The electrical conductivity was calculated from the film thickness and resistance according to the literature^49^.

### Cyclic voltammetry (CV)

Electrochemical properties of all of the samples were investigated by CV using a VersaSTAT3 electrochemical working station in a presence of 5 mM [Fe(CN)6]^3−/4−^ in 0.1 M KCl solution at a 0.1 V/s scan rate.

### UV/vis spectroscopy

PPy and γ-PPy films on ITO were analyzed by UV-vis spectroscopy (Agilent 8453, Agilent Technologies, USA). The absorbance of the irradiated bare ITO with various doses was measured and used as the backgrounds to obtain the absorbance of the γ-PPy films.

### Sterilization test *Escherichia coli* (*E. coli*)

(ATCC 43895) and *Staphylococcus aureus* (*S. aureus*) (ATCC 14458) were prepared as model gram negative and gram positive bacterium, respectively. Bacterial solutions were diluted with 1 mL of LB media to have a density of 1 × 10^7^ for each bacterium. The PPy substrates were immersed in the bacteria-containing LB media and incubated for 24 h at 37 °C with gentle shaking. Then, the media containing the electrodes was exposed to gamma-irradiation at 15, 25, 35, 50, and 75 kGy with a dose rate of 10 kGy/h, respectively. After the sterilization with gamma-ray, the irradiated media (5 µL) was spread onto the LB agar plate and further by incubated for 18 h at 37 °C. Images of the individual plates were acquired using the ChemiDoc imaging systems (ChemiDoc, XRS+, BIO-RAD, Hercules, USA) with 0.428 s exposure time to check the possible colonies on the plates.

### *In vitro* cytocompatibility test

NIH3T3 and C2C12 cells were maintained in DMEM (Hyclone) containing 10% FBS (Hyclone) and antibiotic-antimycotic solutions (Gibco) on tissue culture plate with 5% CO_2_ at 37 °C. PC12 cells were maintained in DMEM medium containing 10% heat-inactivated HS (Gibco), 5% FBS, and 1% antibiotic-antimycotic solution with 5% CO_2_ at 37 °C. Culture media was exchanged every 3 d and cells were passaged with a 0.05% trypsin-EDTA solution (Gibco) at 90% confluence. Prior to cell culture experiments, PC12 cells were cultured on collagen coated tissue culture plate in the differentiation medium (F-12K medium (Gibco) containing 1% heat-inactivated HS, 0.5% FBS, and 1% antibiotic-antimycotic solution) with 100 ng/mL soluble nerve growth factor (NGF) (7 S, murine submaxillary gland, Promega, USA) one week prior to an experiment. For cell seeding, the samples were washed three times with a deionized water and PBS (Gibco) for 1 h. For metabolic activity measurement by the WST-1 assay, polydimethylsiloxane (PDMS) wells (6 mm in inner diameter) were placed on bare electrodes, PPy, and γ-PPy electrodes. Cells were seeded at a density of 5,000 cells into each well. The cells were incubated in the culture medium for 48 h with 5% CO_2_ at 37 °C. After cell culture, the cell viability was quantified by the WST-1 assay according to the manufacturer’s protocol. For individual sample wells, WST-1 reagent solution was added and incubated for 3 h. Absorbance of the solution was measured at 450 nm using a scanning multi-well spectrophotometer (FL600, Bio-Tek, Winooski, Vermont, USA) (*n* = 3).

For immuno-staining, the cells were seeded on substrates at a density of 2 × 10^4^ cells/cm^2^ and cultured for 48 h. After incubation, the samples were fixed in 3.7% paraformaldehyde at room temperature for 20 min. The samples were then washed with DPBS. Cells were permeabilized in DPBS containing 0.1% Triton X-100 for 10 min and incubated in the blocking solution (2% bovine serum albumin (BSA) (Sigma-Aldrich) in DPBS (Gibco)) for 2 min. For *F*-actin staining, the samples were incubated at room temperature for 20 min in Aelxa-fluor 488-conjugated phalloidin (Invitrogen) (1:300 in blocking solution). The samples were washed with DPBS and then incubated in 4′-6-diamidino-2-phenylindole (DAPI (Invitrogen), 1:2500 in PBS) solution to stain nuclei. Fluorescence images were acquired using florescence microscope (DMI3000B, Leica, Germany).

### Statistical analysis

All tests were performed at least in triplicate and data were presented as the mean ± standard deviation (SD) unless otherwise noted. Statistical significance was examined by one-way analysis of variance (ANOVA) with a Tukey’s post-hoc comparison of the means using Origin software. A p-value less than 0.05 was considered to be statistically significant.

## Electronic supplementary material

Supplementary information
