# Supplementary material for: Effective gamma-ray sterilization and characterization of conductive polypyrrole biomaterials
Source: Sci Rep. 2018 Feb 27;8:3721. doi: 10.1038/s41598-018-22066-6 (PMC5829140; doi:10.1038/s41598-018-22066-6)
Supplement: Supplementary file 1 — Supplementary information [file 41598_2018_22066_MOESM1_ESM.doc]

**Supporting Information**

Effective gamma-ray sterilization and characterization of conductive polypyrrole biomaterials

Semin Kim^1+^, Jin-Oh Jeong^1,2+^, Sanghun Lee^3^, Jong-Seok Park^2^, Hui-Jeong Gwon^2^, Sung In Jeong^2^, John George Hardy^4,5^, Youn-Mook Lim^2*^, and Jae Young Lee^1,6*^

*^1^* School of Materials Science and Engineering, Gwangju Institute of Science and Technology, Gwangju 61005, Republic of Korea

*^2^* Research Division for Industry & Environment, Advanced Radiation Technology

Institute, Korea Atomic Energy Research Institute (KAERI), 29 Gumgugil, Jeongeup,

56212, Republic of Korea

*^3^* Materials Science and Engineering Concentration, GIST College, Gwangju, 61005, Republic of Korea

*^4^* Department of Chemistry, Lancaster University, Lancaster, Lancashire, LA1 4YB, United Kingdom

*^5^* Materials Science Institute, Lancaster University, Lancaster, Lancashire, LA1 4YB, United Kingdom

*^6^* Department of Biomedical Science and Engineering, Gwangju Institute of Science and Technology, Gwangju 61005, Republic of Korea

*500-712, Republic of Korea*

+ These authors contributed equally.

* E-mail: jaeyounglee@gist.ac.kr (JYL); ymlim71@kaeri.re.kr (YML)

**Contents:**

**Figure S1.** Potential-time curves for the galvanostatic electrodeposition of PPy/pTS films in 0.15 M Pyyrrole, 0.1 M pTS solution at1 mA/cm2, 100 mC.

**Figure S2.** Surface morphology of irradiated-PPy-ITO films with the different irradiation dose by Scanning Electron Microscopy (SEM); (a) PPy, (b) γ-PPy 15, (c) γ-PPy 25, (d) γ-PPy 35, (e) γ-PPy 50, and (f) γ-PPy 75

**Figure S3.** Photographs of the PPy and γ-PPy electrodes before and after the Scotch tape detachment test.

**Figure S4.** Bode plots of autoclaved PPy electrodes

**Figure S5.** Stereo-microscope images of PPy and autoclaved-PPy electrodes. An arrow indicates a wrinkles on PPy film. An arrow head indicates a cracks.

**Figure S6.** Peak potential separation and capacitance from cyclic voltammograms.

**Figure S7.** Schematic diagram of sterilization assay on gamma-irradiated PPy films.

.
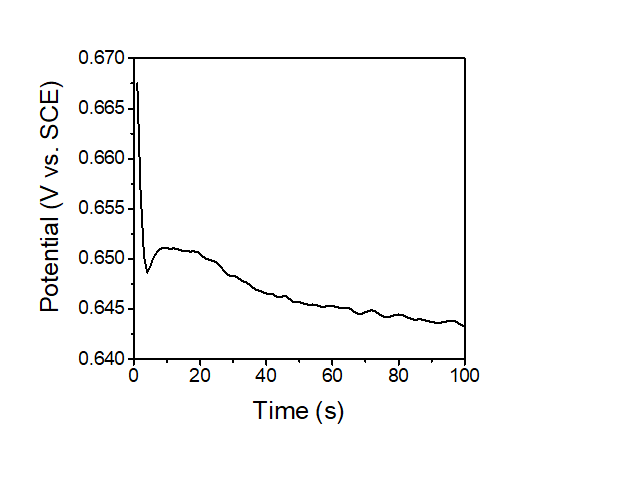


**Figure S1.** A potential time curve during the galvanostatic electrodeposition of PPy/pTS films in the monomer solution (0.15 M pyrrole and 0.1 M pTS) at 1 mA/cm^2^ and 100 mC.


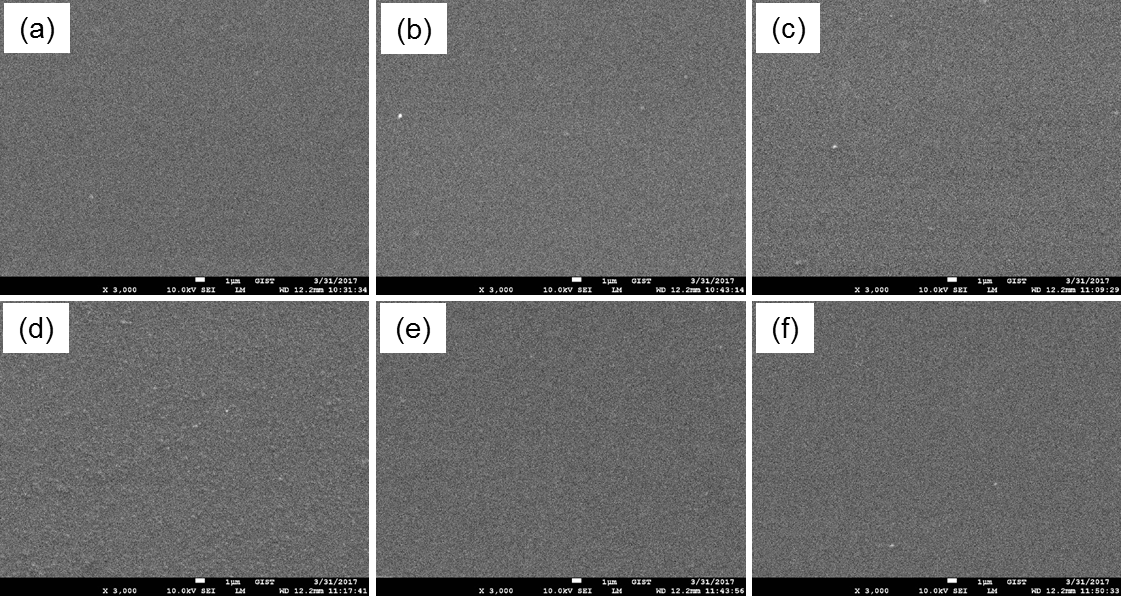


**Figure S2.** Scanning electron micrographs of the γ-PPy; (a) PPy, (b) γ-PPy 15, (c) γ-PPy 25, (d) γ-PPy 35, (e) γ-PPy 50, and (f) γ-PPy 75.


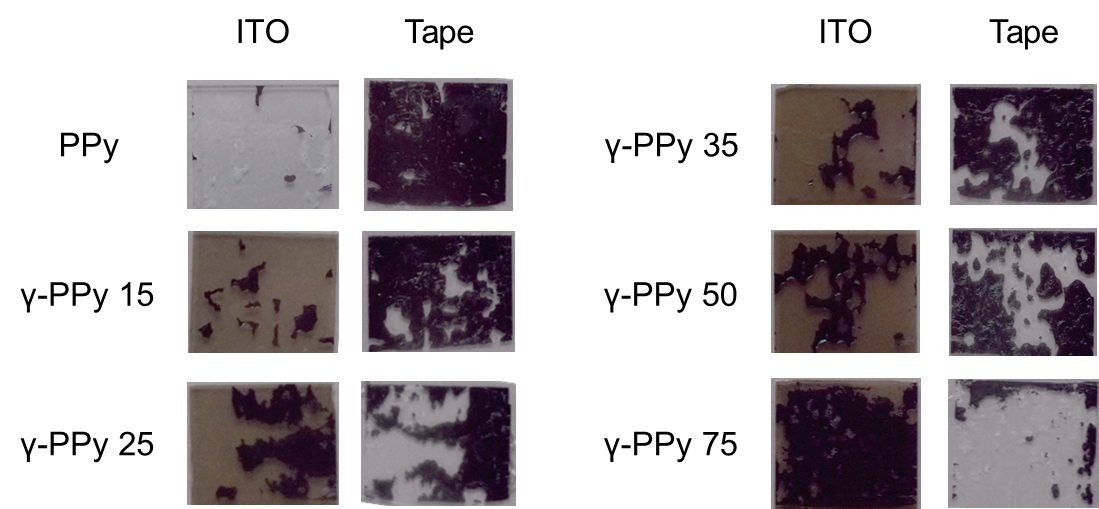


**Figure S3.** Photographs of the PPy and γ-PPy electrodes before and after the Scotch tape detachment test.

**
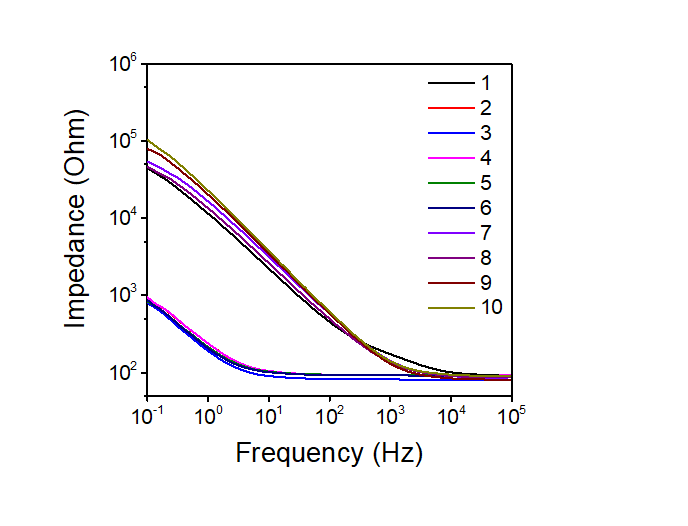
**

**Figure S4.** Bode plots of autoclaved PPy electrodes. Ten samples were measured.


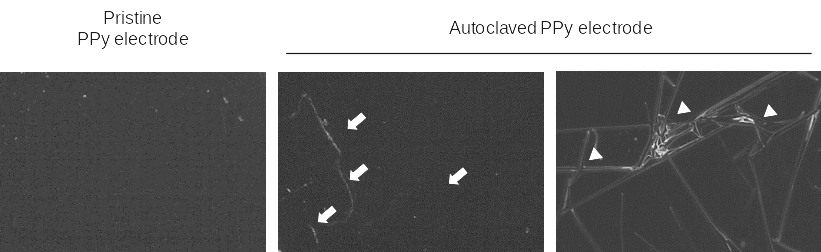


**Figure S5.** Stereo-microscope images of PPy and autoclaved-PPy electrodes. An arrow indicates wrinkles on PPy films. An arrow head indicates cracks.

**
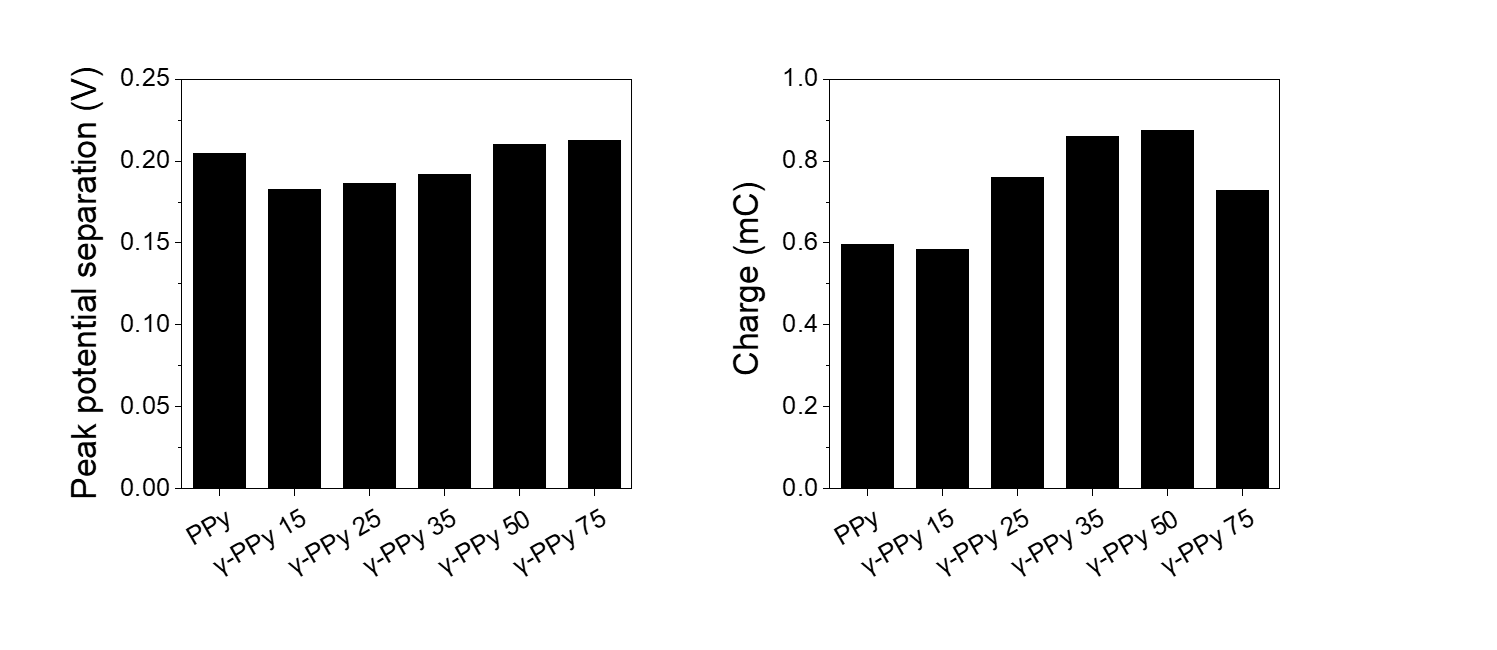
**

**Figure S6.** Peak potential separation and capacitance from cyclic voltammogram.


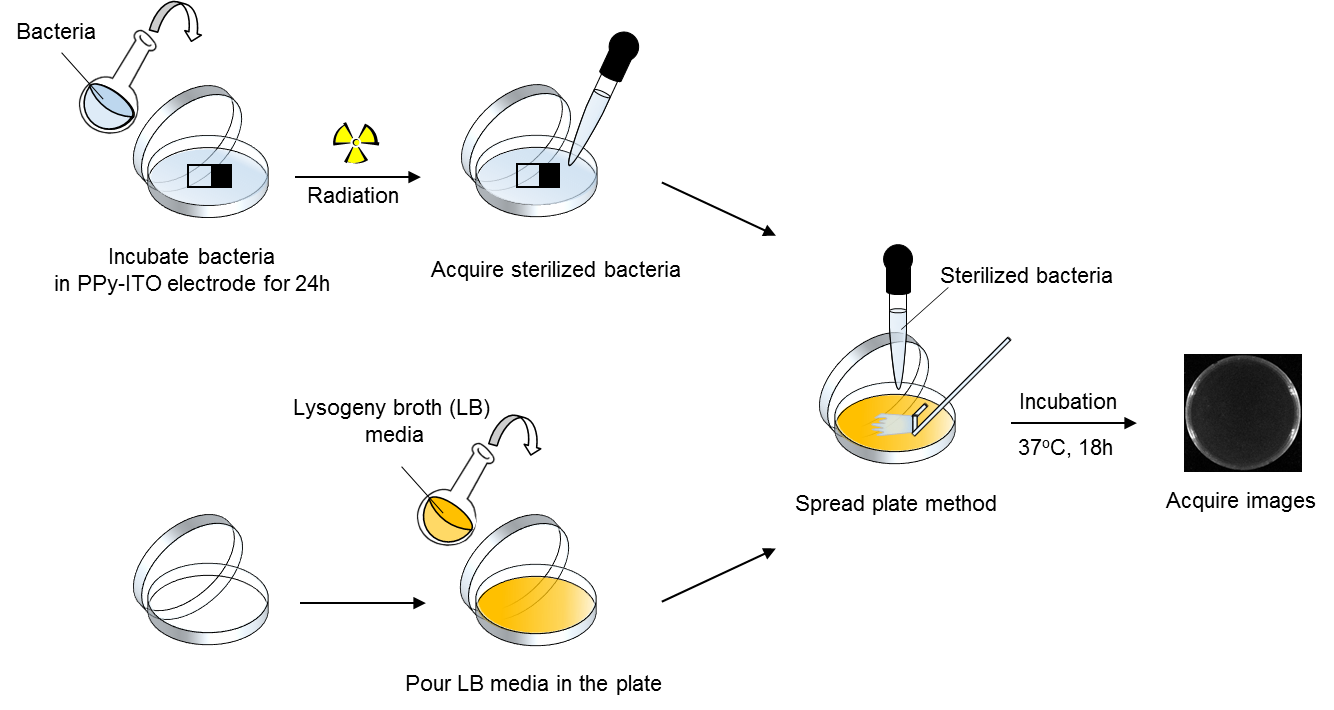


**Figure S7.** Schematic diagram of sterilization assay on gamma-irradiated PPy films.

This material is available free of charge via the Internet at http://pubs.acs.org.
